# Retraction Note: Transgenic mice overexpressing the ALS-linked protein Matrin 3 develop a profound muscle phenotype

**DOI:** 10.1186/s40478-017-0502-0

**Published:** 2017-12-13

**Authors:** Christina Moloney, Sruti Rayaprolu, John Howard, Susan Fromholt, Hilda Brown, Matt Collins, Mariela Cabrera, Colin Duffy, Zoe Siemienski, Dave Miller, Maurice S. Swanson, Lucia Notterpek, David R. Borchelt, Jada Lewis

**Affiliations:** 1 Center for Translational Research in Neurodegenerative Disease, University of Florida, Gainesville, FL USA; 2 Department of Neuroscience, University of Florida, Gainesville, FL USA; 3 Department of Molecular Genetics and Microbiology, Center for NeuroGenetics and the Genetics Institute, University of Florida, College of Medicine, Gainesville, FL USA; 4 McKnight Brain Institute, Department of Neuroscience, University of Florida, Gainesville, FL USA

## Retraction Note: acta neuropathol commun (2016) 4: 122. https://doi.org/10.1186/s40478-016-0393-5

The authors are retracting this article. The article describes mice expressing wild-type human MATR3. However, since publication the authors have become aware that all of the lines of mice described express human MATR3 containing the F115C mutation. Transgenic mice expressing wild-type and mutant Matrin were created simultaneously in their laboratory and, at a crucial stage of generating the DNA for embryo injection, as confirmed by an investigation by the University of Florida, the DNA preparations were accidentally mislabelled. All of the founders created were mosaic, requiring extensive breeding to isolate stable lines. Mice mislabelled as expressing wild-type MATR3 were the first to produce lines that stably transmitted the transgene and thus were the first to be characterized. However, as lines of mice that were mislabelled as expressing the mutant (F115C) MATR3 were ultimately established, the data began to suggest that an error had been made. Sequence analysis of amplified tail DNA from mice descended from the lines reported in the article have revealed that they express the F115C variant. The data described are therefore an accurate description of the pathology of mice that express the F115C variant of MATR3, but not of mice expressing wild-type MATR3. The authors are preparing a new manuscript reporting data from both mice expressing the F115C variant of MATR3 and mice expressing wild-type MATR3.

